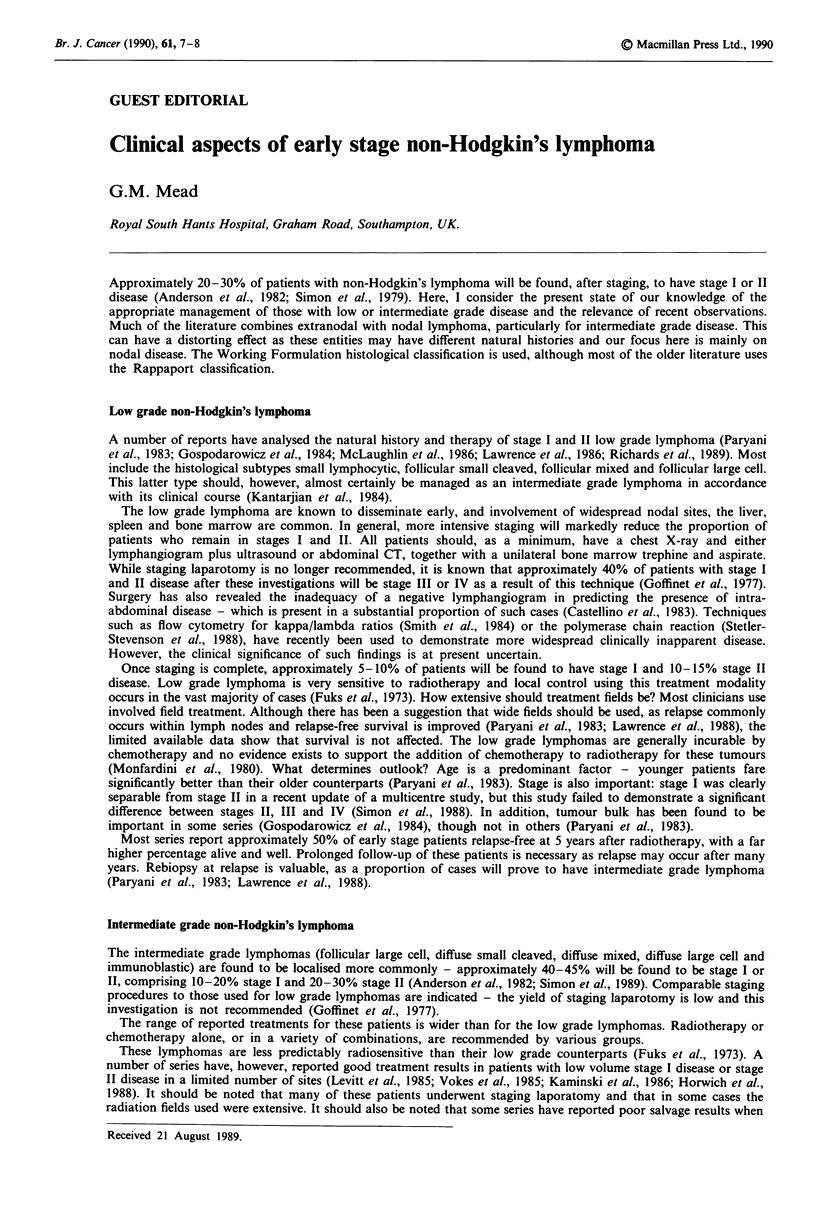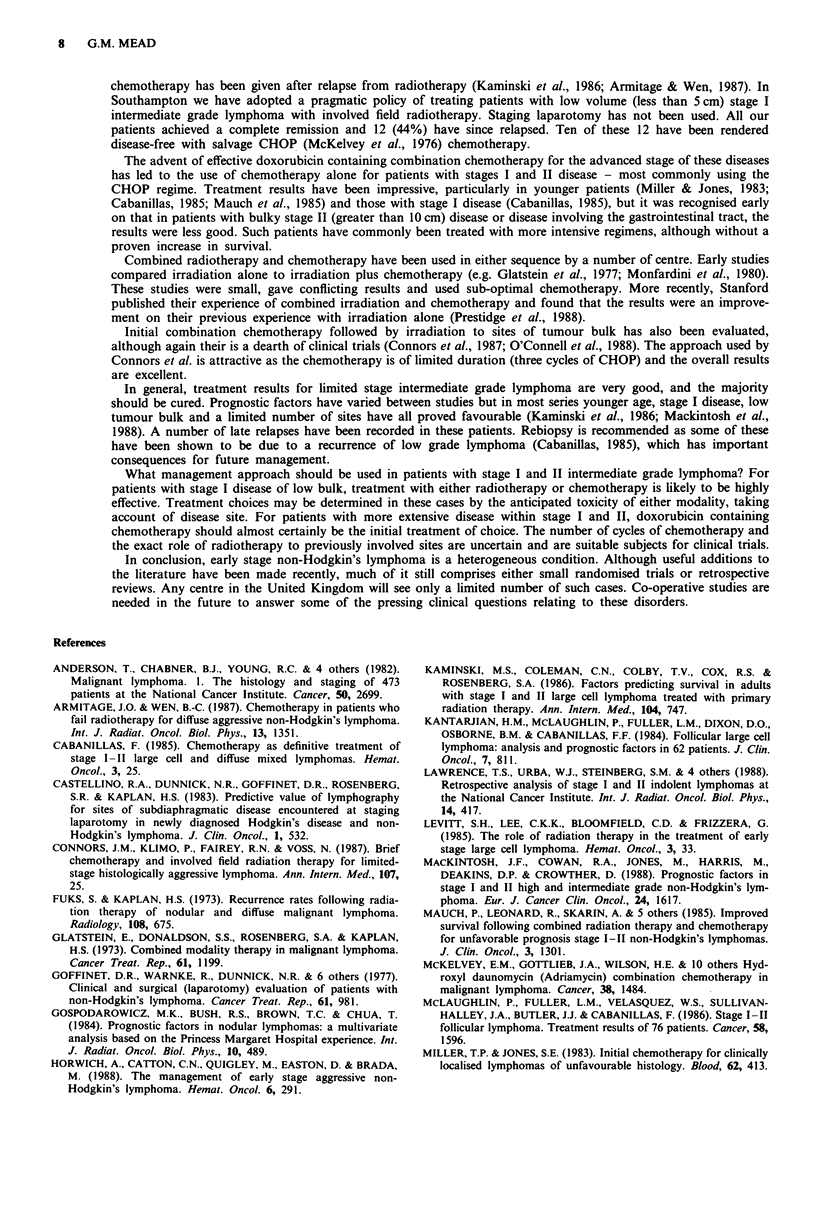# Clinical aspects of early stage non-Hodgkin's lymphoma.

**DOI:** 10.1038/bjc.1990.3

**Published:** 1990-01

**Authors:** G. M. Mead

**Affiliations:** Royal South Hants Hospital, Southampton, UK.


 © Macmillan Press Ltd., 1990

GUEST EDITORIAL

### Clinical aspects of early stage non-Hodgkin's lymphoma

G.M. Mead

*Royal South Hants Hospital, Graham Road, Southampton, UK.*

Approximately 20–30% of patients with non-Hodgkin's lymphoma will be found, after staging, to have stage I or II disease (Anderson *et al.*, 1982; Simon *et al.*, 1979). Here, I consider the present state of our knowledge of the appropriate management of those with low or intermediate grade disease and the relevance of recent observations. Much of the literature combines extranodal with nodal lymphoma, particularly for intermediate grade disease. This can have a distorting effect as these entities may have different natural histories and our focus here is mainly on nodal disease. The Working Formulation histological classification is used, although most of the older literature uses the Rappaport classification.

### Low grade non-Hodgkin's lymphoma

A number of reports have analysed the natural history and therapy of stage I and II low grade lymphoma (Paryani *et al.*, 1983; Gospodarowicz *et al.*, 1984; McLaughlin *et al.*, 1986; Lawrence *et al.*, 1986; Richards *et al.*, 1989). Most include the histological subtypes small lymphocytic, follicular small cleaved, follicular mixed and follicular large cell. This latter type should, however, almost certainly be managed as an intermediate grade lymphoma in accordance with its clinical course (Kantarjian *et al.*, 1984).

The low grade lymphoma are known to disseminate early, and involvement of widespread nodal sites, the liver, spleen and bone marrow are common. In general, more intensive staging will markedly reduce the proportion of patients who remain in stages I and II. All patients should, as a minimum, have a chest X-ray and either lymphangiogram plus ultrasound or abdominal CT, together with a unilateral bone marrow trephine and aspirate. While staging laparotomy is no longer recommended, it is known that approximately 40% of patients with stage I and II disease after these investigations will be stage III or IV as a result of this technique (Goffinet *et al.*, 1977). Surgery has also revealed the inadequacy of a negative lymphangiogram in predicting the presence of intra-abdominal disease – which is present in a substantial proportion of such cases (Castellino *et al.*, 1983). Techniques such as flow cytometry for kappa/lambda ratios (Smith *et al.*, 1984) or the polymerase chain reaction (Stetler-Stevenson *et al.*, 1988), have recently been used to demonstrate more widespread clinically inapparent disease. However, the clinical significance of such findings is at present uncertain.

Once staging is complete, approximately 5–10% of patients will be found to have stage I and 10–15% stage II disease. Low grade lymphoma is very sensitive to radiotherapy and local control using this treatment modality occurs in the vast majority of cases (Fuks *et al.*, 1973). How extensive should treatment fields be? Most clinicians use involved field treatment. Although there has been a suggestion that wide fields should be used, as relapse commonly occurs within lymph nodes and relapse-free survival is improved (Paryani *et al.*, 1983; Lawrence *et al.*, 1988), the limited available data show that survival is not affected. The low grade lymphomas are generally incurable by chemotherapy and no evidence exists to support the addition of chemotherapy to radiotherapy for these tumours (Monfardini *et al.*, 1980). What determines outlook? Age is a predominant factor – younger patients fare significantly better than their older counterparts (Paryani *et al.*, 1983). Stage is also important: stage I was clearly separable from stage II in a recent update of a multicentre study, but this study failed to demonstrate a significant difference between stages II, III and IV (Simon *et al.*, 1988). In addition, tumour bulk has been found to be important in some series (Gospodarowicz *et al.*, 1984), though not in others (Paryani *et al.*, 1983).

Most series report approximately 50% of early stage patients relapse-free at 5 years after radiotherapy, with a far higher percentage alive and well. Prolonged follow-up of these patients is necessary as relapse may occur after many years. Rebiopsy at relapse is valuable, as a proportion of cases will prove to have intermediate grade lymphoma (Paryani *et al.*, 1983; Lawrence *et al.*, 1988).

### Intermediate grade non-Hodgkin's lymphoma

The intermediate grade lymphomas (follicular large cell, diffuse small cleaved, diffuse mixed, diffuse large cell and immunoblastic) are found to be localised more commonly – approximately 40–45% will be found to be stage I or II, comprising 10–20% stage I and 20–30% stage II (Anderson *et al.*, 1982; Simon *et al.*, 1989). Comparable staging procedures to those used for low grade lymphomas are indicated – the yield of staging laparotomy is low and this investigation is not recommended (Goffinet *et al.*, 1977).

The range of reported treatments for these patients is wider than for the low grade lymphomas. Radiotherapy or chemotherapy alone, or in a variety of combinations, are recommended by various groups.

These lymphomas are less predictably radiosensitive than their low grade counterparts (Fuks *et al.*, 1973). A number of series have, however, reported good treatment results in patients with low volume stage I disease or stage II disease in a limited number of sites (Levitt *et al.*, 1985; Vokes *et al.*, 1985; Kaminski *et al.*, 1986; Horwich *et al.*, 1988). It should be noted that many of these patients underwent staging laporatomy and that in some cases the radiation fields used were extensive. It should also be noted that some series have reported poor salvage results when

Received 21 August 1989.

chemotherapy has been given after relapse from radiotherapy (Kaminski *et al.*, 1986; Armitage & Wen, 1987). In Southampton we have adopted a pragmatic policy of treating patients with low volume (less than 5 cm) stage I intermediate grade lymphoma with involved field radiotherapy. Staging laparotomy has not been used. All our patients achieved a complete remission and 12 (44%) have since relapsed. Ten of these 12 have been rendered disease-free with salvage CHOP (McKelvey *et al.*, 1976) chemotherapy.

The advent of effective doxorubicin containing combination chemotherapy for the advanced stage of these diseases has led to the use of chemotherapy alone for patients with stages I and II disease – most commonly using the CHOP regime. Treatment results have been impressive, particularly in younger patients (Miller & Jones, 1983; Cabanillas, 1985; Mauch *et al.*, 1985) and those with stage I disease (Cabanillas, 1985), but it was recognised early on that in patients with bulky stage II (greater than 10 cm) disease or disease involving the gastrointestinal tract, the results were less good. Such patients have commonly been treated with more intensive regimens, although without a proven increase in survival.

Combined radiotherapy and chemotherapy have been used in either sequence by a number of centre. Early studies compared irradiation alone to irradiation plus chemotherapy (e.g. Glatstein *et al.*, 1977; Monfardini *et al.*, 1980). These studies were small, gave conflicting results and used sub-optimal chemotherapy. More recently, Stanford published their experience of combined irradiation and chemotherapy and found that the results were an improvement on their previous experience with irradiation alone (Prestidge *et al.*, 1988).

Initial combination chemotherapy followed by irradiation to sites of tumour bulk has also been evaluated, although again their is a dearth of clinical trials (Connors *et al.*, 1987; O'Connell *et al.*, 1988). The approach used by Connors *et al.* is attractive as the chemotherapy is of limited duration (three cycles of CHOP) and the overall results are excellent.

In general, treatment results for limited stage intermediate grade lymphoma are very good, and the majority should be cured. Prognostic factors have varied between studies but in most series younger age, stage I disease, low tumour bulk and a limited number of sites have all proved favourable (Kaminski *et al.*, 1986; Mackintosh *et al.*, 1988). A number of late relapses have been recorded in these patients. Rebiopsy is recommended as some of these have been shown to be due to a recurrence of low grade lymphoma (Cabanillas, 1985), which has important consequences for future management.

What management approach should be used in patients with stage I and II intermediate grade lymphoma? For patients with stage I disease of low bulk, treatment with either radiotherapy or chemotherapy is likely to be highly effective. Treatment choices may be determined in these cases by the anticipated toxicity of either modality, taking account of disease site. For patients with more extensive disease within stage I and II, doxorubicin containing chemotherapy should almost certainly be the initial treatment of choice. The number of cycles of chemotherapy and the exact role of radiotherapy to previously involved sites are uncertain and are suitable subjects for clinical trials.

In conclusion, early stage non-Hodgkin's lymphoma is a heterogeneous condition. Although useful additions to the literature have been made recently, much of it still comprises either small randomised trials or retrospective reviews. Any centre in the United Kingdom will see only a limited number of such cases. Co-operative studies are needed in the future to answer some of the pressing clinical questions relating to these disorders.

### References

ANDERSON, T., CHABNER, B.J., YOUNG, R.C. & 4 others (1982). Malignant lymphoma. 1. The histology and staging of 473 patients at the National Cancer Institute. *Cancer*, **50**, 2699.

ARMITAGE, J.O. & WEN, B.-C. (1987). Chemotherapy in patients who fail radiotherapy for diffuse aggressive non-Hodgkin's lymphoma. *Int. J. Radiat. Oncol. Biol. Phys.*, **13**, 1351.

CABANILLAS, F. (1985). Chemotherapy as definitive treatment of stage I–II large cell and diffuse mixed lymphomas. *Hemat. Oncol.*, **3**, 25.

CASTELLINO, R.A., DUNNICK, N.R., GOFFINET, D.R., ROSENBERG, S.R. & KAPLAN, H.S. (1983). Predictive value of lymphography for sites of subdiaphragmatic disease encountered at staging laparotomy in newly diagnosed Hodgkin's disease and non-Hodgkin's lymphoma. *J. Clin. Oncol.*, **1**, 532.

CONNORS, J.M., KLIMO, P., FAIREY, R.N. & VOSS, N. (1987). Brief chemotherapy and involved field radiation therapy for limited-stage histologically aggressive lymphoma. *Ann. Intern. Med.*, **107**, 25.

FUKS, S. & KAPLAN, H.S. (1973). Recurrence rates following radiation therapy of nodular and diffuse malignant lymphoma. *Radiology*, **108**, 675.

GLATSTEIN, E., DONALDSON, S.S., ROSENBERG, S.A. & KAPLAN, H.S. (1973). Combined modality therapy in malignant lymphoma. *Cancer Treat. Rep.*, **61**, 1199.

GOFFINET, D.R., WARNKE, R., DUNNICK, N.R. & 6 others (1977). Clinical and surgical (laparotomy) evaluation of patients with non-Hodgkin's lymphoma. *Cancer Treat. Rep.*, **61**, 981.

GOSPODAROWICZ, M.K., BUSH, R.S., BROWN, T.C. & CHUA, T. (1984). Prognostic factors in nodular lymphomas: a multivariate analysis based on the Princess Margaret Hospital experience. *Int. J. Radiat. Oncol. Biol. Phys.*, **10**, 489.

HORWICH, A., CATTON, C.N., QUIGLEY, M., EASTON, D. & BRADA, M. (1988). The management of early stage aggressive non-Hodgkin's lymphoma. *Hemat. Oncol.* **6**, 291.

KAMINSKI, M.S., COLEMAN, C.N., COLBY, T.V., COX, R.S. & ROSENBERG, S.A. (1986). Factors predicting survival in adults with stage I and II large cell lymphoma treated with primary radiation therapy. *Ann. Intern. Med.*, **104**, 747.

KANTARJIAN, H.M., MCLAUGHLIN, P., FULLER, L.M., DIXON, D.O., OSBORNE, B.M. & CABANILLAS, F.F. (1984). Follicular large cell lymphoma: analysis and prognostic factors in 62 patients. *J. Clin. Oncol.*, **7**, 811.

LAWRENCE, T.S., URBA, W.J., STEINBERG, S.M. & 4 others (1988). Retrospective analysis of stage I and II indolent lymphomas at the National Cancer Institute. *Int. J. Radiat. Oncol. Biol. Phys.*, **14**, 417.

LEVITT, S.H., LEE, C.K.K., BLOOMFIELD, C.D. & FRIZZERA, G. (1985). The role of radiation therapy in the treatment of early stage large cell lymphoma. *Hemat. Oncol.*, **3**, 33.

MACKINTOSH, J.F., COWAN, R.A., JONES, M., HARRIS, M., DEAKINS, D.P. & CROWTHER, D. (1988). Prognostic factors in stage I and II high and intermediate grade non-Hodgkin's lymphoma. *Eur. J. Cancer Clin. Oncol.*, **24**, 1617.

MAUCH, P., LEONARD, R., SKARIN, A. & 5 others (1985). Improved survival following combined radiation therapy and chemotherapy for unfavorable prognosis stage I–II non-Hodgkin's lymphomas. *J. Clin. Oncol.*, **3**, 1301.

MCKELVEY, E.M., GOTTLIEB, J.A., WILSON, H.E. & 10 others Hydroxyl daunomycin (Adriamycin) combination chemotherapy in malignant lymphoma. *Cancer*, **38**, 1484.

MCLAUGHLIN, P., FULLER, L.M., VELASQUEZ, W.S., SULLIVAN-HALLEY, J.A., BUTLER, J.J. & CABANILLAS, F. (1986). Stage I–II follicular lymphoma. Treatment results of 76 patients. *Cancer*, **58**, 1596.

MILLER, T.P. & JONES, S.E. (1983). Initial chemotherapy for clinically localised lymphomas of unfavourable histology. *Blood*, **62**, 413.